# A Diagnosis of Maternal 22q Duplication and Mosaic Deletion following Prenatal Cell-Free DNA Screening

**DOI:** 10.1155/2023/9127430

**Published:** 2023-11-20

**Authors:** Melissa A. Hicks, Emilie Lalonde, Jessica Zoladz, Bernard Gonik, Salah Ebrahim

**Affiliations:** ^1^DMC University Laboratories & Wayne State University School of Medicine, Detroit, MI 48201, USA; ^2^Wayne State University School of Medicine, Detroit, MI 48201, USA; ^3^London Health Sciences Center, London, Canada; ^4^Natera, Inc., Austin, TX, USA; ^5^Spectrum Health, Grand Rapids, MI, USA

## Abstract

Concurrent microduplication and microdeletion of the chromosome 22q11.2 region are a rarely reported phenomenon. We describe a case of germline 22q11.21 microduplication syndrome with concurrent mosaic 22q11.2 deletion in a pregnant patient, identified by chromosomal microarray and FISH after noninvasive prenatal genetic screening (cfDNA) results discordant with family history. The patient was referred to maternal-fetal medicine (MFM) at 14 weeks' gestation secondary to an SNP-based cfDNA result of a suspected maternal 22q11.2 deletion and a fetal risk of 1 in 2 for 22q11.2 deletion syndrome. The patient reported a similar cfDNA result in a previous pregnancy; however postnatal chromosomal microarray on that child identified an atypical 22q11.21 microduplication. We report the maternal chromosomal microarray findings of a germline 726 kb 22q11.21 duplication and a mosaic 1.33 Mb 22q11.2 deletion and highlight the copy number variant data generated by cfDNA in this unique case. This family adds to the limited literature of concurrent 22q11.2 microduplication and microdeletion carriers.

## 1. Introduction

Prenatal cell-free DNA screening (cfDNA) has revolutionized aneuploidy screening in the United States [1]. The American College of Obstetricians and Gynecologists (ACOG) continues to recommend that all patients should be counseled in each pregnancy about options for testing for fetal aneuploidies, including screening (biochemical, ultrasound, and cfDNA) and diagnostic tests. They do not recommend screening for microdeletions with cfDNA due to a lack of clinical validation [2].

By comparison, the American College of Medical Genetics & Genomics (ACMG) recommends cfDNA for all singleton and twin pregnancies if screening for fetal trisomies 21, 13, & 18 is desired. They also suggest that 22q11.2 deletion syndrome be offered to all patients after a discussion about benefits and limitations in the context of shared decision-making. Per their 2022 guideline, there is insufficient evidence to recommend routine screening for copy number variants (CNVs) other than 22q11.2, though they do acknowledge that there will be families for whom other CNVs could be offered based on pregnancy or family history, after thorough pretest counseling [3].

The recurrent 22q11.2 deletion is the most common microdeletion worldwide, with an incidence of 1 in 1000 fetuses [4] and 1 in 3000 to 1 in 6000 live births. It is caused by nonallelic homologous recombination (NAHR) between nearby low copy repeats (LCRs). Typically, NAHR causes deletions between LCRs A–D (∼3 Mb) and LCRs A-B (∼1.5 Mb), but additional LCRs can result in atypical or nested microdeletions or reciprocal microduplications [5]. Fetal screening for 22q11.2 deletion syndrome may enable prenatal detection of congenital heart defects, birth at a center with an intensive care unit, and timely treatment for neonatal hypocalcemia and immunodeficiency, which improves outcomes [6, 7].

In this report, we describe a pregnant patient identified as likely having 22q11.2 deletion syndrome, herself, by SNP-based cfDNA in two different pregnancies. Peripheral blood chromosomal microarray identified a germline 726 kb 22q11.21 duplication (LCRs B–D) and a mosaic 1.33 Mb 22q11.2 deletion (LCRs A-B). We highlight the data generated by cfDNA in this unique case and the importance of follow-up of unusual cfDNA findings.

## 2. Case Report

A 33-year-old gravida 6 para 1 patient presented at 14 + 0 weeks gestation for MFM consultation, secondary to abnormal SNP-based cfDNA results. Obstetric history was significant for three spontaneous pregnancy losses and one ectopic pregnancy. Prenatal cell-free DNA screening was ordered by her primary obstetric care provider at 9 + 6 weeks' gestation. The fetal fraction was 8.9%. The results revealed a suspected maternal deletion, putting the male fetus at a 1 in 2 risk for 22q11.2 deletion syndrome. The patient's pregnancy history was significant for a similar cfDNA result in a previous pregnancy two years prior. Records confirmed and reported fetal fraction at 10.8%. The patient declined amniocentesis in both pregnancies.

Patient consent was obtained to review her previous child's records. That child, a son then ∼10 months old, was born at 40 + 3 weeks' gestation via caesarean section for fetal heart rate abnormalities. Postnatal complications included small size for gestational age (SGA), meconium aspiration, ventricular septal defect, ECMO therapy, hearing loss, and minor facial dysmorphisms including triangular face, bitemporal narrowing, epicanthus inversus, flat, depressed nasal bridge and broad, anteverted nares (similar in appearance to father's nose), low-set and posteriorly rotated ears, high-arched palate, and micrognathia. Genetic consultation and chromosomal microarray were performed while in the neonatal intensive care unit. Microarray identified an atypical nested 22q11.21 duplication spanning 725.83 kb, between LCRs B-D, including the *CRKL* gene. Outpatient follow-up with a pediatric geneticist and genetic counselor emphasized that 22q11.21 duplications may be inherited from an apparently unaffected parent, especially since the expression is highly variable [8, 9]. The patient was interested in testing herself for duplication, and insurance authorization was initiated; however, neither the patient nor her son presented for follow-up in the 8 months between the son's appointment and the high-risk cfDNA result in the subsequent (i.e., current) pregnancy.

**Figure 1:**
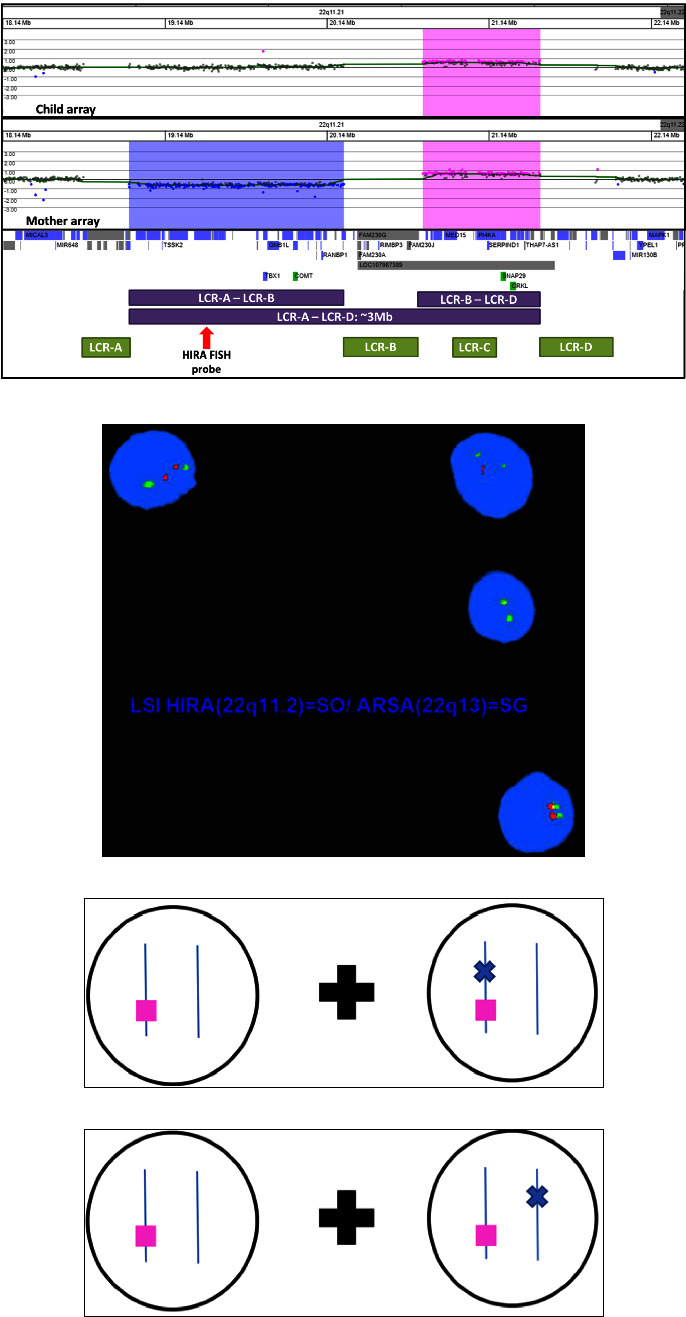
(a) Chromosomal microarray results demonstrating the 726 kb duplication in the 10-month-old son (top panel) and the co-occurrence of both the 1.33 Mb deletion and the 725 kb duplication in the mother (middle panel). The approximate locations of the LCRs and HIRA FISH probe are shown in the bottom panel. (b) Maternal peripheral blood FISH for 22q11.2 confirming mosaicism for the deletion (75% showing deletion). The red signal hybridizes to the *HIRA* gene on 22q11.2, located between LCRs A and B, while the green signal hybridizes to a control locus, *ARSA* on 22q13 (Vysis LSI DiGeorge/VCFS probe). (c) Possible configuration of maternal duplication and mosaic deletion *in cis*; pink square = duplication, blue *X* = deletion. (d) Possible configuration of maternal duplication and mosaic deletion *in trans;* pink square = duplication, blue *X* = deletion.

After discussion and coordination between the MFM, the referring obstetric provider, and the cfDNA laboratory's genetic counselor, the patient underwent peripheral blood chromosomal microarray analysis. This revealed not only the same 725.83 kb atypical nested 22q11.21 duplication seen in her 10-month-old son but also a mosaic 1.33 Mb 22q11.2 deletion between LCRs A-B. Subsequent FISH analysis using the Vysis LSI DiGeorge/VCFS probe, encompassing the *HIRA* gene, was performed on an uncultured blood sample to confirm mosaicism for the 22q11.2 deletion; there was no FISH probe available for the duplication. The hybridization produced a deletion pattern in 74.2% of nuclei and a normal pattern in 25.8% of nuclei, consistent with mosaicism; however, *cis* versus *trans* configuration could not be determined by these assays (Figure 1).

**Figure 2:**
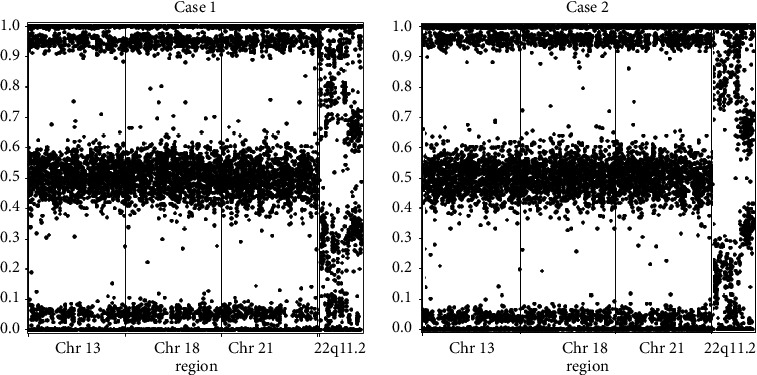
SNP plots from the two pregnancies show a maternal 22q11.2 deletion and possible maternal duplication in the same region. The *x*-axis shows the relative position of SNPs along the analyzed chromosomes. The *y*-axis shows the relative amounts of the alleles. The shifting of the upper, lower, and middle bands in the 22q11.2 region indicates a deletion is present. As expected, the pattern is similar between the two cases. Note: this graphical representation of data does not in any way describe the functioning of Natera's technology.

Following the diagnosis of maternal CNVs, the local laboratory and clinical teams contacted the cfDNA laboratory. Internal review demonstrated evidence for the maternal deletion and duplication confirmed by microarray; however, the latter was not a validated nor reportable finding (Figure 2). A review of records indicated that the patient had never self-reported a personal history of learning difficulties, cardiac defects, seizures, or kidney disease during routine obstetrical visits. Further review of her records indicates she is “blind in her left eye” and reported an alcohol use disorder. The patient was referred to a medical geneticist for full dysmorphic and developmental evaluation; however, she did not attend that appointment. There was no record of a cardiology consult or echocardiogram. Further conversation with the patient by phone (MAH) revealed that she felt she had “learning problems.”

The risk for 22q11.2 deletion and duplication in the current pregnancy was again raised following the maternal findings; however, this risk was uncertain as somatic or gonadal mosaicism could not be ruled out. The patient was offered amniocentesis for fetal diagnosis but declined. After the delivery of an apparently healthy child, the pediatrician ordered chromosomal microarray at our institution, which revealed a normal male profile. Follow-up with pediatric genetics has not been pursued by the family to this point.

## 3. Discussion

The phenotypic consequence of 22q11.21 LCR A-D duplications is highly heterogeneous, ranging from asymptomatic to more severe phenotypes such as congenital heart defects, velopharyngeal insufficiency with or without cleft palate, hearing loss, growth and/or developmental delay, learning difficulties, autism spectrum disorder, behavioral issues, and vision issues [10]. Prenatally, cardiac and renal anomalies (associated with duplication of the *CRKL* gene), cleft palate (associated with duplication of the *SPECC1L* gene), increased nuchal translucency, and mild skeletal anomalies may be observed. While there is no consistent pattern of facial features, hypertelorism, broad/flat nose, micrognathia, abnormally formed ears, ear pits or tags, epicanthal folds, and downslanting palpebral fissures have been reported among individuals with duplications from regions A-B, B–D, C-D, and F–H; this is consistent with the findings in our patient's affected son. In ∼60% of cases, duplications were inherited from an apparently unaffected parent [10].

The clinical significance of smaller, nested duplications within the 22q11.2 region, including the B-D duplication seen in the family described here, is less clear. These duplications are associated with highly variable expression and low penetrance; most individuals are asymptomatic, and the duplication may be found incidentally. Case-control studies have been conflicting, but it has been postulated that the B-D region includes the critical genes involved in the neuropsychiatric phenotypes, thus predisposing individuals to developmental delay, autism spectrum disorder, and behavioral problems [11, 12]. Further studies are needed to fully understand the consequences of B-D duplications and to identify additional factors affecting phenotypic expression.

While 22q11.2 microdeletion syndrome is a common microdeletion syndrome, cases of mosaicism are infrequently reported. A recent study, however, estimates that the frequency of mosaicism in 22q11.2 microdeletion syndrome can be as high as 28.2% [13]. Phenotypic variability does not appear to correlate with the level of mosaicism. Though rare, 22q11.21 microduplication has been found in combination with a 22q11.2 microdeletion. An individual with an LCR A-B deletion in 70% of cells by FISH, with an inherited LCR B–D duplication, has been reported; that patient presented with submucosal cleft palate, recurrent urinary infections, severe myopia, learning difficulties, stenosis of the left pulmonary artery, low levels of B-lymphocytes, immunoglobulin (Ig)G and IgA, panic, anxiety, and problems with social interaction. In this case, the mosaic 22q11.2 deletion was thought to be a postzygotic event. Paternal family members with only 22q11.2 duplication had variable findings including aortic aneurysm orofacial clefting, minor motor difficulties, and recurrent ear infections [14]. In another case report, a dichorionic-diamniotic twin with mosaicism for both deletion and duplication of the 1.5 Mb region of 22q11.2 flanked by LCRs A-B was described. That patient presented with truncus arteriosus type III and interrupted aortic arch type B, significant neonatal hypocalcemia, feeding difficulties, recurrent infections, and global developmental delay. Circular swirls of hypo-hyperpigmentation were consistent with mosaicism [15]. It is important to note that patients with 22q11.21 duplication and/or 22q11.2 deletion can have vastly different presentations and physical exam findings, even within the same family. Whether the patient reported in this case has the deletion and duplication in *cis* or *trans* is not known. Any future pregnancies will be at increased risk for both the deletion (unknown but elevated) and the duplication (50%).

Massively parallel signature sequencing (MPSS)-based methods of cfDNA have historically had a positive predictive value for 22q11.2 deletions of about 20% [16, 17]. Single-nucleotide polymorphism (SNP)-based cfDNA is also capable of screening 22q11.2 deletion syndrome and currently reports a sensitivity of 83.3% (CI 51.6–97.9), specificity of 99.95%, and a positive predictive value of 52.6% (CI 28.9–75.6) for 22q11.2 deletion syndrome, including the typical LCR A–D deletion and nested deletions between LCRs A-B, A–C, and B–D ranging in size from 0.73–2 Mb [18]. While not validated nor reported, the cfDNA platform could potentially detect the microduplication in our patient (personal communication). Though currently the sensitivity and specificity of CNVs detected by cfDNA is lower than for whole chromosome aneuploidies, this is an emerging area of expanded clinical cfDNA application.

In conclusion, we present a case of concurrent microdeletion and duplication of 22q11.2, uniquely and incidentally identified after abnormal prenatal cell-free DNA screening (cfDNA) in two consecutive pregnancies. This highlights the importance of diagnostic testing to clarify findings after high-risk or unusual cfDNA results.

## Data Availability

The data used to support the findings of this study are included within the article.

## References

[1] Benn P., Borrell A., Chiu R. W. (2015). Position statement from the chromosome abnormality screening committee on behalf of the board of the international society for prenatal diagnosis. *Prenatal Diagnosis*.

[2] Rose N. C., Kaimal A. J., Dugoff L., Norton M. E. (2020). Screening for fetal chromosomal abnormalities: ACOG practice bulletin, number 226. *Obstetrics and Gynecology*.

[3] Dungan J. S., Klugman S., Darilek S. (2023). Noninvasive prenatal screening (NIPS) for fetal chromosome abnormalities in a general-risk population: an evidence-based clinical guideline of the American College of Medical Genetics and Genomics (ACMG). *Genetics in Medicine*.

[4] Grati F. R., Molina Gomes D., Ferreira J. C. (2015). Prevalence of recurrent pathogenic microdeletions and microduplications in over 9500 pregnancies. *Prenatal Diagnosis*.

[5] McDonald-McGinn D. M., Sullivan K. E., Marino B. (2015). 2 deletion syndrome. *Nature Reviews Disease Primers*.

[6] Quartermain M. D., Hill K. D., Goldberg D. J. (2019). Prenatal diagnosis influences preoperative status in neonates with congenital heart disease: an analysis of the society of thoracic surgeons congenital heart surgery database. *Pediatric Cardiology*.

[7] Cheung E. N., George S. R., Andrade D. M., Chow E. W., Silversides C. K., Bassett A. S. (2014). Neonatal hypocalcemia, neonatal seizures, and intellectual disability in 22q11. 2 deletion syndrome. *Genetics in Medicine*.

[8] Bartik L. E., Hughes S. S., Tracy M. (2022). 22q11.2 duplications: expanding the clinical presentation. *American Journal of Medical Genetics, Part A*.

[9] Manno G. C., Segal G. S., Yu A. (2021). Genotypic and phenotypic variability of 22q11. 2 microdeletions–an institutional experience. *AIMS molecular science*.

[10] Mary L., Lavillaureix A., Perrot A. (2022). Prenatal phenotype of 22q11 micro-duplications: a systematic review and report on 12 new cases. *European Journal of Medical Genetics*.

[11] Coe B. P., Witherspoon K., Rosenfeld J. A. (2014). Refining analyses of copy number variation identifies specific genes associated with developmental delay. *Nature Genetics*.

[12] Woodward K. J., Stampalia J., Vanyai H. (2019). Atypical nested 22q11. 2 duplications between LCR 22B and LCR 22D are associated with neurodevelopmental phenotypes including autism spectrum disorder with incomplete penetrance. *Molecular genetics and genomic medicine*.

[13] Halder A., Jain M., Kalsi A. K. (2018). Mosaicism in 22q11. 2 microdeletion syndrome. *Journal of Clinical and Diagnostic Research*.

[14] Blennow E., Lagerstedt K., Malmgren H., Sahlén S., Schoumans J., Anderlid B. M. (2008). Concurrent microdeletion and duplication of 22q11. 2. *Clinical Genetics*.

[15] Dempsey M. A., Schwartz S., Waggoner D. J. (2007). Mosaicism del (22)(q11. 2q11. 2)/dup (22)(q11. 2q11. 2) in a patient with features of 22q11. 2 deletion syndrome. *American Journal of Medical Genetics, Part A*.

[16] Lefkowitz R. B., Tynan J. A., Liu T. (2016). Clinical validation of a noninvasive prenatal test for genomewide detection of fetal copy number variants. *American Journal of Obstetrics and Gynecology*.

[17] Perinatal Quality (2015). Nipt/cell free dna screening predictive value calculator. https://www.perinatalquality.org/vendors/nsgc/nipt/.

[18] Dar P., Jacobsson B., Clifton R. (2022). Cell-free DNA screening for prenatal detection of 22q11. 2 deletion syndrome. *American Journal of Obstetrics and Gynecology*.

